# Carbon dioxide fluxes in Alpine grasslands at the Nivolet Plain, Gran Paradiso National Park, Italy 2017–2023

**DOI:** 10.1038/s41597-024-03374-1

**Published:** 2024-06-21

**Authors:** Angelica Parisi, Francesca Avogadro di Valdengo, Ilaria Baneschi, Alice Baronetti, Maria Virginia Boiani, Maurizio Catania, Sara Lenzi, Marta Magnani, Pietro Mosca, Antonello Provenzale, Brunella Raco, Gianna Vivaldo, Mariasilvia Giamberini

**Affiliations:** 1 Istituto di Geoscienze e Georisorse, IGG-CNR, Via Moruzzi 1, 56124 Pisa, Italy; 2 Joint CNR-ENI Research Centre on the Arctic Cryosphere “Aldo Pontremoli”, Nanotec-CNR, via Monteroni, 73100 Lecce, Italy; 3 National Biodiversity Future Centre, 90133 Palermo, Italy; 4 Centro Interdipartimentale sui Rischi Naturali in Ambiente Montano e Collinare, NatRisk, Via Verdi 8, 10124 Turin, Italy; 5 Istituto Nazionale di Fisica Nucleare, INFN, Via P. Giuria 1, 10125 Torino, Italy; 6 Istituto di Geoscienze e Georisorse, IGG-CNR, Via Valperga Caluso 35, 10125 Turin, Italy; 7 Present Address: Currently at University of Genoa, DIBRIS - Department of Informatics, Bioengineering, Robotics and Systems Engineering, Genova, Italy; 8 Present Address: Currently at Chester University, Department of Biological Sciences, CH14BJ Parkgate Road, Chester, UK

**Keywords:** Carbon cycle, Climate sciences

## Abstract

We introduce a georeferenced dataset of Net Ecosystem Exchange (NEE), Ecosystem Respiration (ER) and meteo-climatic variables (air and soil temperature, air relative humidity, soil volumetric water content, pressure, and solar irradiance) collected at the Nivolet Plain in Gran Paradiso National Park (GPNP), western Italian Alps, from 2017 to 2023. NEE and ER are derived by measuring the temporal variation of CO_2_ concentration obtained by the enclosed chamber method. We used a customised portable non-steady-state dynamic flux chamber, paired with an InfraRed Gas Analyser (IRGA) and a portable weather station, measuring CO_2_ fluxes at a number of points (around 20 per site and per day) within five different sites during the snow-free season (June to October). Sites are located within the same hydrological basin and have different geological substrates: carbonate rocks (site CARB), gneiss (GNE), glacial deposits (GLA, EC), alluvial sediments (AL). This dataset provides relevant and often missing information on high-altitude mountain ecosystems and enables new comparisons with other similar sites, modelling developments and validation of remote sensing data.

## Background & Summary

Earth’s changing climate is significantly affecting mountain ecosystems^1^. Temperature rise and modification of precipitation patterns lead to glacier retreat, reduction of snow cover, alteration of the water cycle, and impacts on living organisms and biogeochemical cycles. In particular, climate change can affect the structure and functioning of mountain ecosystems, particularly for what concerns the natural carbon cycle. Previous research indicated that natural grasslands act as a net carbon sink^2–4^. However, the carbon fluxes and carbon storage capacity of these ecosystems are likely to change in response to climate warming, particularly in high-mountain areas that are more susceptible to temperature rise^5,6^. Quantifying the carbon fluxes at the soil-vegetation-atmosphere interface in high-mountain ecosystems, and simultaneously measuring meteo-climatic and environmental variables, is an essential source of information for investigating what are the main drivers of carbon fluxes and understanding the response of CO_2_ fluxes to climate change.

To this aim, in 2017 the Institute of Geosciences and Earth Resources of the National Research Council of Italy (IGG-CNR) established an Alpine Critical Zone Observatory (CZO@NIVOLET) in the north-western Italian Alps (Nivolet Plain, Gran Paradiso National Park).

The dataset presented here is the result of data collection using portable non-steady-state flux chambers and weather stations. The dataset contains measurements collected at individual points within the study sites approximately every 10–15 days during the snow-free period in seven years of fieldwork, from 2017 to 2023.

The non-steady-state flux chamber is a classical method used for estimating gas fluxes, in particular greenhouse gases such as CO_2_ and CH_4_, from different types of interfaces, including bare soil^7,8^ and natural ecosystems^9,10^.

Part of the data presented here were already published as average values of point-measurements for each site and each sampling date, and are freely available in the IGG-CNR-CZO community of the Zenodo repository^11–13^.

In a related research article titled “*Drivers of carbon fluxes in Alpine tundra: a comparison of three empirical model approaches*”^14^ multi-regression models were developed for Gross Primary Production (GPP, defined as GPP = NEE - ER) and Ecosystem Respiration (ER) using average values for each site and each sampling date from years 2017, 2018, and 2019. Further investigations, based on the above-mentioned average values and additional data from CZO@NIVOLET, have also been discussed in “C*arbon dioxide exchanges in an alpine tundra ecosystem (Gran Paradiso National Park, Italy): A comparison of results from different measurement and modelling approaches*”^15^ and “*Spatial and temporal variability of carbon dioxide fluxes in the Alpine Critical Zone: The case of the Nivolet Plain, Gran Paradiso National Park, Italy*”^16^.

In this manuscript, we present and make freely available the complete dataset of point-measurements, which were previously analysed only as averages over site and sampling date. Moreover, this dataset includes data from the 2023 field campaigns, which have never been used nor published in any form before.

This dataset enables new modelling and analysis efforts by the scientific community. It can be used for spatio-temporal analysis of CO_2_ fluxes in Alpine ecosystems, for comparisons with CO_2_ fluxes from other environments, and for validating models developed by using remote sensing data. It can also be used for diagnostic purposes in the analysis of the dependence of CO_2_ fluxes on climate drivers.

In addition to sharing the complete dataset with the research community, this manuscript provides a comprehensive description of the CZO@NIVOLET site’s methodology for data collection and processing using the portable flux chamber method. This description encompasses each step of the process, from the instruments’ calibration at the dedicated laboratory in a controlled environment to the calculation of CO_2_ fluxes, reported as μmolCO_2_ m^−2^ s^−1^. Furthermore, we emphasise that the CZO@NIVOLET site remains actively investigated. This manuscript then provides guidance to the understanding and utilisation of present and forthcoming data generated in this study site which will be as well updated within the IGG-CNR-CZO community of the Zenodo repository.

## Methods

### Study site

The CZO@NIVOLET was installed in 2017 and is located within the boundaries of the Gran Paradiso National Park (GPNP). It is part of the Critical Zone Exploration Network (CZEN, https://www.czen.org/content/nivolet-czo), a global network investigating processes in the Critical Zone, which is defined as the dynamic living skin of the Earth that extends from the top of the vegetative canopy through the soil and down to fresh bedrock and the bottom of the groundwater^17^. This research site also belongs to both the European eLTER (https://elter-ri.eu/elter-ri) and ICOS ERIC (https://www.icos-cp.eu) Research Infrastructures (RI).

**Fig. 1:**
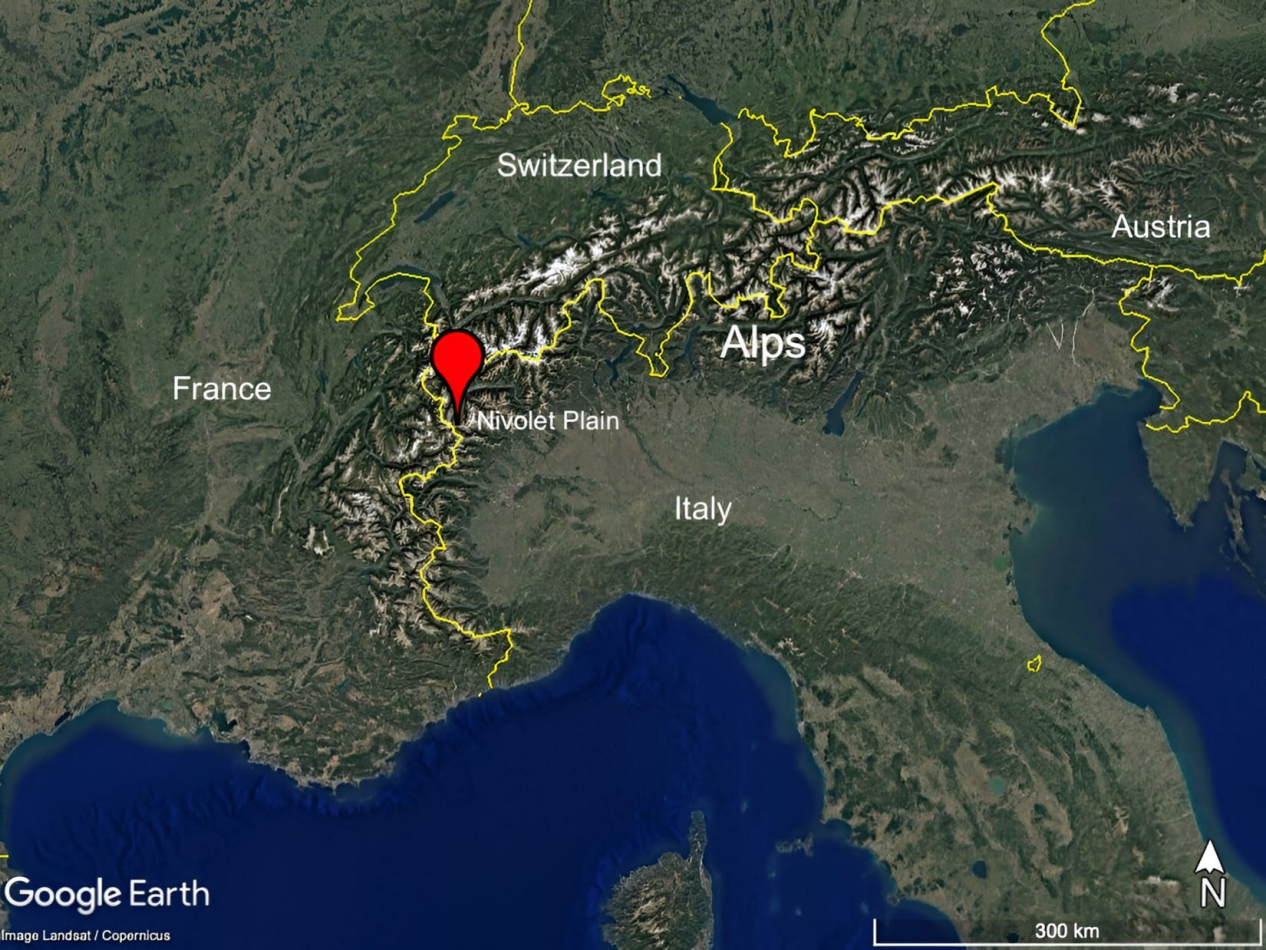
Location of the CZO@NIVOLET. The Nivolet Plain (45°28′42.96″N 7°08′31.92″E) is located in the north-western Italian Alps. The image was acquired by Landsat/Copernicus, sourced and modified from Google Earth.

The GPNP was established in 1922 for the preservation of the Alpine ibex (*Capra ibex*) and the conservation of high-altitude mountain ecosystems. Encompassing an area of 720 km^2^, the park features a wide range of ecosystems, including lower elevation Alpine woods, as well as high-altitude grasslands and Alpine tundra, rock cliffs, and glaciers above the treeline. The Nivolet Plain (Fig. 1) is a glacial valley that ranges in elevation from approximately 2300 m a.s.l. in the northeast to around 2700 m a.s.l. in the southwest.

The underlying bedrock is composed of gneisses, dolostones and marbles from the Gran Paradiso Massif, as well as calcschists with serpentinites and metabasites from the Piedmont-Ligurian zone^18^.

Daily records of precipitation (mm), minimum, maximum, and mean temperature (°C) from the Lago Agnel weather station are freely available (CC BY-NC-SA 4.0) at Arpa Piemonte portal (https://www.arpa.piemonte.it/rischi_naturali/snippets_arpa_graphs/dati_giornalieri_meteo/?statid=PIE-001073-900-1996-10-10&param=P). According to such data, over the time span 2017–2023, the average daily minimum temperature from June to October was 6.11 °C, the average daily maximum temperature was 13.0 °C, and the average daily precipitation was 2.9 mm. During winter the soil is typically covered with a thick layer of snow.

The Nivolet Plain is home to Alpine natural grasslands that support a diverse array of species within the *Caricion curvulae* climax vegetation community^19^. Dominant species found in the grasslands include *Carex curvula* All., *Alopecurus gerardi* Vill., *Gnaphalium supinum* L., and *Leontodon helveticus* Mérat. In the investigation sites, also *Geum montanum*, *Trifolium alpinum*, *Pulsatilla alpina*, and *Silene acaulis* are commonly found. The plants in these high-altitude grasslands experience rapid development from late June to late October, with canopy heights reaching a maximum of 0.2 metres.

During summer, grasslands are grazed by both domestic and wild ungulates. Wild ungulates (ibex and chamois) are censused every year. In 2022, the population density in the GPNP was counted to be 2687 ibex individuals and 6346 chamois individuals (GPNP, unpublished data, see also^20^), while there are no quantitative census data on roe deer, red deer, and wild boar. These latter, however, are typically found at lower altitudes than those considered in our study. Regarding domestic ungulates, from the beginning of July until mid-September, approximately 110 cows along with around 20 sheep, and goats are brought for grazing in this area^19^. Grazing is conducted in a controlled manner, with animals predominantly grazing in areas adjacent to the barn (located at 45°29'13.7“N 7°08'27.6“E) and throughout the lower regions of the Nivolet valley (see https://www.pastoralp.eu/homepage/ for more information).

**Fig. 2:**
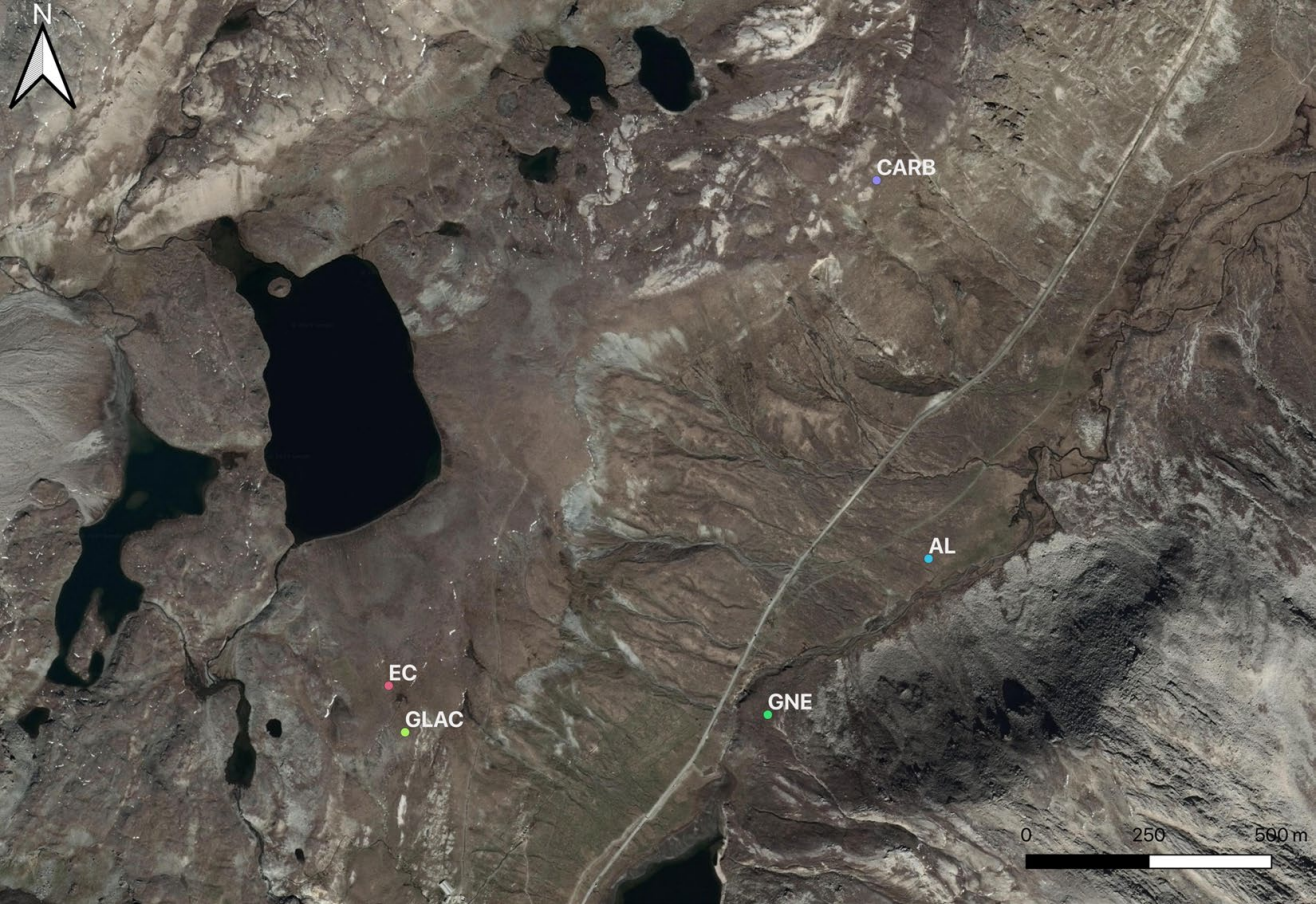
Location of the five measurement sites. Soils developed on carbonate rocks (CARB), gneiss (GNE), glacial deposits (GLAC & EC) and alluvial sediments (AL). Made with Qgis software (v.3.16 Hannover, QGIS Development Team, 2021, www.qgis.org) (Map data ©2015 Google).

The five measurement sites at the Nivolet Plain are located within the same hydrological basin, and each site has an area between ~ 500 to ~ 900 square metres. Three of the five selected sites are on the orographic left flank bordering the Nivolet Plain: one on carbonate rocks (site named CARB in the dataset, at 2750–2760 m a.s.l.) and two on glacial deposits (site named GLA, at 2740–2750 m a.s.l. and site named EC, at 2750–2760 m a.s.l.). One site lies on the orographic right flank of the Plain, on soils developed on gneiss (site named GNE, at about 2580–2600 m a.s.l.). One site is on alluvial soil at the Plain floor (site named AL, 2740–2750 m a.s.l.). The location of the five sites is shown in Fig. 2. Mean coordinates of the five study sites are reported in Table 1.

**Table 1:** Mean coordinates of the five study sites.

Site	MEAN Lon	MEAN Lat	MEAN Elevation
AL	7.15345	45.49324	2490–2500 m a.s.l.
CARB	7.15210	45.50016	2750–2760 m a.s.l.
EC	7.13937	45.49091	2750–2760 m a.s.l.
GLAC	7.13979	45.49006	2740–2750 m a.s.l.
GNE	7.14926	45.49038	2580–2600 m a.s.l.

Mean coordinates were calculated by averaging the latitude and longitude of individual measurement points. First column: site name; second and third columns: mean longitude and mean latitude (WGS84); fourth column: mean elevation.

In 2020, D’Amico *et al*.^21^ published the soil types map of the Aosta Valley, encompassing the region that includes our study area. Besides, our research group conducted soil profile samplings in locations proximate to and with similar geological and geomorphological attributes of the five study sites. Some physical and chemical characteristics of soil profiles are briefly described here^22,23^.

Since 2020, data on aboveground vegetation biomass at the EC site have become available. These values, calculated as averages from individual samples, are detailed in Table 2.

**Table 2:** Mean biomass values for aboveground vegetation at EC site.

Year	Biomass	N. of Samples	Date of Sampling
2020	0.23	20	08/08/2020
2021	0.197	16	14/07/2021
2023	0.14	15	26/07/2023

Mean biomass values were calculated by averaging individual vegetation samples. The biomass samples were obtained by harvesting 0.25 m x 0.25 m aboveground vegetation plots at the maximum growing season. The samples were then dried to constant weight at 60 °C for 48 hours and weighed. These measurements were conducted at the ICOS associated station Nivolet (ICOS code IT-Niv), which corresponds to the EC site. The procedure was conducted in accordance with the guidelines outlined here^32^ for ICOS associated stations and followed a modified protocol derived from the ICOS procedures described here^33^. These, along with other data, are freely accessible here^34^ upon registration on the ICOS data portal (ICOS CCBY4 Data Licence). First column: sampling year in yyyy format; second column: mean biomass, expressed in kilograms of dry matter per square metre (kgDM m^−2^); third column: total number of biomass samples collected for averaging; fourth column: date of sampling in dd/mm/yyyy format.

### Flux chamber measurements

**Fig. 3:**
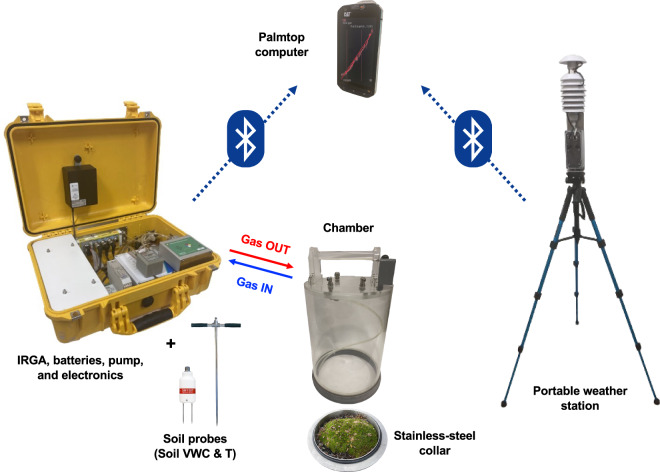
Portable instrumentation setup. The yellow case on the left contains the IRGA (Infrared Gas Analyzer), batteries, pump, and electronics. The IRGA is connected to the flux chamber through two RILSAN® tubing pipes (gas IN and gas OUT), each measuring 1.8 m in length and having an internal diameter of 4 mm and an external diameter of 6 mm. The gas sampling line is protected by two types of filters: (1) a 50 mm diameter PTFE membrane filter with a pore size of 0.45 μm, and (2) a 25 mm diameter PTFE membrane filter with a pore size of 0.2 μm. These filters are permeable to gases and water vapour but are impermeable to liquid water and dust particles. The soil volumetric water content is recorded using a TDR (Time-Domain Reflectometry) soil sensor. The soil temperature is recorded using a Pt100 soil thermometer. All data are recorded at 1 Hz during the measurement. Air relative humidity, air temperature, and solar irradiance are measured by a portable weather station (thermohygrometer and pyranometer) mounted on a tripod at a height of 1.5 metres above the ground (on the right). An Android device (palmtop computer) connected via Bluetooth serves as an interface for managing the measurement, displaying, and storing the data.

In the summer of 2017, surveys were carried out to select measurement sites, assess instrumental setups, and determine the CO_2_ flux ranges essential for laboratory calibration of portable flux chamber systems. This initial phase was characterised by few measurement campaigns. Starting from 2018, the frequency of these campaigns increased, establishing a regular schedule of measurements approximately every 10–15 days throughout the vegetative season. The final instrumentation setup is shown in Fig. 3.

NEE and ER were measured using the non-steady state dynamic flux chamber method. The chamber, placed over the vegetated soil, isolates a volume of air where the concentration of CO_2_ increases or decreases according to the dominant process at the soil-vegetation-atmosphere interface. In the presence of sunlight, if photosynthesis captures CO_2_ faster than its release due to respiration, the CO_2_ concentration inside the chamber decreases. If respiration is dominant over photosynthesis, the CO_2_ concentration increases. CO_2_ concentration inside the flux chamber is measured over a specific time interval; the flux is computed by interpolating the curve of CO_2_ concentration versus time, as explained further in the text and discussed in detail here^24^.

At each point, a stainless-steel collar was inserted for about 1 cm into the soil a few minutes before the measurement, assuring no leakage. Before placing the flux chamber, RGB (Red, Green, Blue) images were taken from a nadir perspective, aiming at monitoring the vegetation within the collar area. These images are freely available in the IGG-CNR-CZO community of the Zenodo repository^25^.

The flux chamber was then placed on the collar to isolate a confined air volume (headspace). This created a closed system where the CO_2_ concentration inside the chamber changes during the measurement because of CO_2_ absorption by plants through photosynthesis and/or emission through respiration by autotrophs (i.e., plants) and heterotrophs (i.e., microbial communities in the soil).

Air from the headspace of the chamber was pumped at a constant flow rate of 3 l/min into an Infrared Gas Analyzer (IRGA, either model LI-840 or LI-850 CO_2_/H_2_O Analyzer; LI-COR Biosciences, Lincoln, NE, USA) through a 1.8-metres-long RILSAN® tubing. The sampled air was then reinjected into the chamber. The reinjection tube ended with a 0.40-metres-long coiled and pierced RILSAN® tube, which ensured good mixing of the reinjected air sample within the chamber. Before and after each measurement, the entire apparatus (including the chamber, tubing, and IRGA) was vented until the ambient CO_2_ concentration was recorded and its concentration was stable for a few seconds.

The CO_2_ concentration inside the flux chamber was measured for about 90 seconds. CO_2_ concentration versus time was recorded at 1 Hz frequency using the custom Android app FluxManager2 (West Systems S.r.l., freely available on Google Play Store) which was installed on a palmtop computer connected to the instrument via Bluetooth. Upon completion of the measurements, a text file containing all the data, including meteo-climatic and environmental variables (see below), was generated in the internal memory of the Android device.

The concentration curve was interpolated linearly over a period of about 60 seconds to calculate the rate of change of CO_2_ concentration over time (ppm s^−1^). The interpolation was done using the custom FluxRevision software (West Systems S.r.l). The initial 10–15 seconds (cleaning time), and the final, potentially non-linear part of the curve were excluded from the interpolation.

Measurements were conducted at various times throughout the day, ranging from 10:00 to 18:00^9^, and covered different meteorological conditions, in order to capture the natural meteorological variability^26^. For each measurement campaign and for each site, measurements were replicated at 15–20 different points within the site, randomly chosen to sample the small-scale flux variability. Previous analysis has shown that a minimum of 15 measurement points is generally sufficient to represent the spatial variability at these sites^14^.

**Fig. 4:**
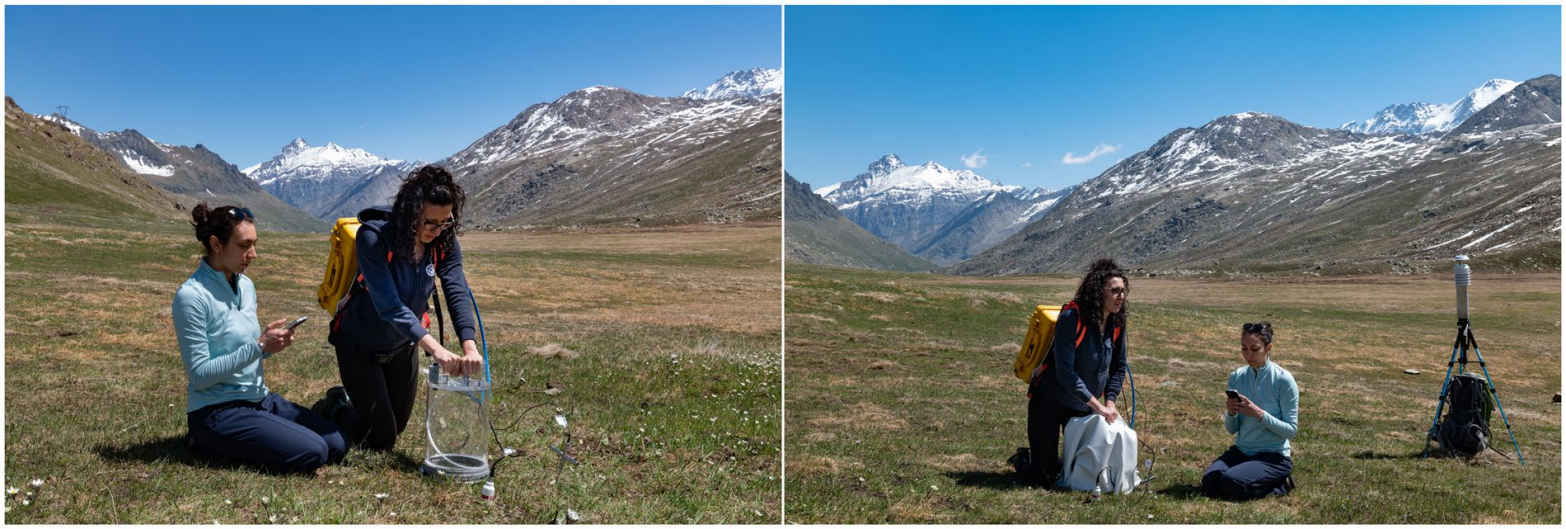
Measurement procedure. The standard measurement cycle consists of two consecutive measurements at each point. The first measurement is conducted under natural light conditions using the transparent chamber (left) to determine the NEE. The second measurement is performed using the shaded chamber (right) to determine the ER. All individuals in figures have provided explicit consent for their images to be openly published.

Figure 4 shows a typical measurement cycle, which included two consecutive measurements at each point: the first was performed under ambient light, using the transparent chamber to estimate NEE, while the second measurement was performed using the same chamber shaded with a cloth to estimate ER in the absence of photosynthesis. A similar procedure was applied in previous works on similar environments^26,27^. This process was repeated at all points, requiring approximately 2 hours to cover an entire site. Notice that in 2020, owing to the restrictions imposed by the pandemics, the number of measurement campaigns had to be much reduced.

NEE and ER fluxes in μmolCO_2_ m^−2^ s^−1^ were estimated from the slope of the linear regression of headspace CO_2_ concentration over time (ppm s^−1^) using a laboratory calibration curve that relates pre-determined CO_2_ fluxes (in the range of fluxes expected in the field) with the corresponding measured slopes of the CO_2_ vs time linear regression (see the section “Technical Validation”).

**Fig. 5:**
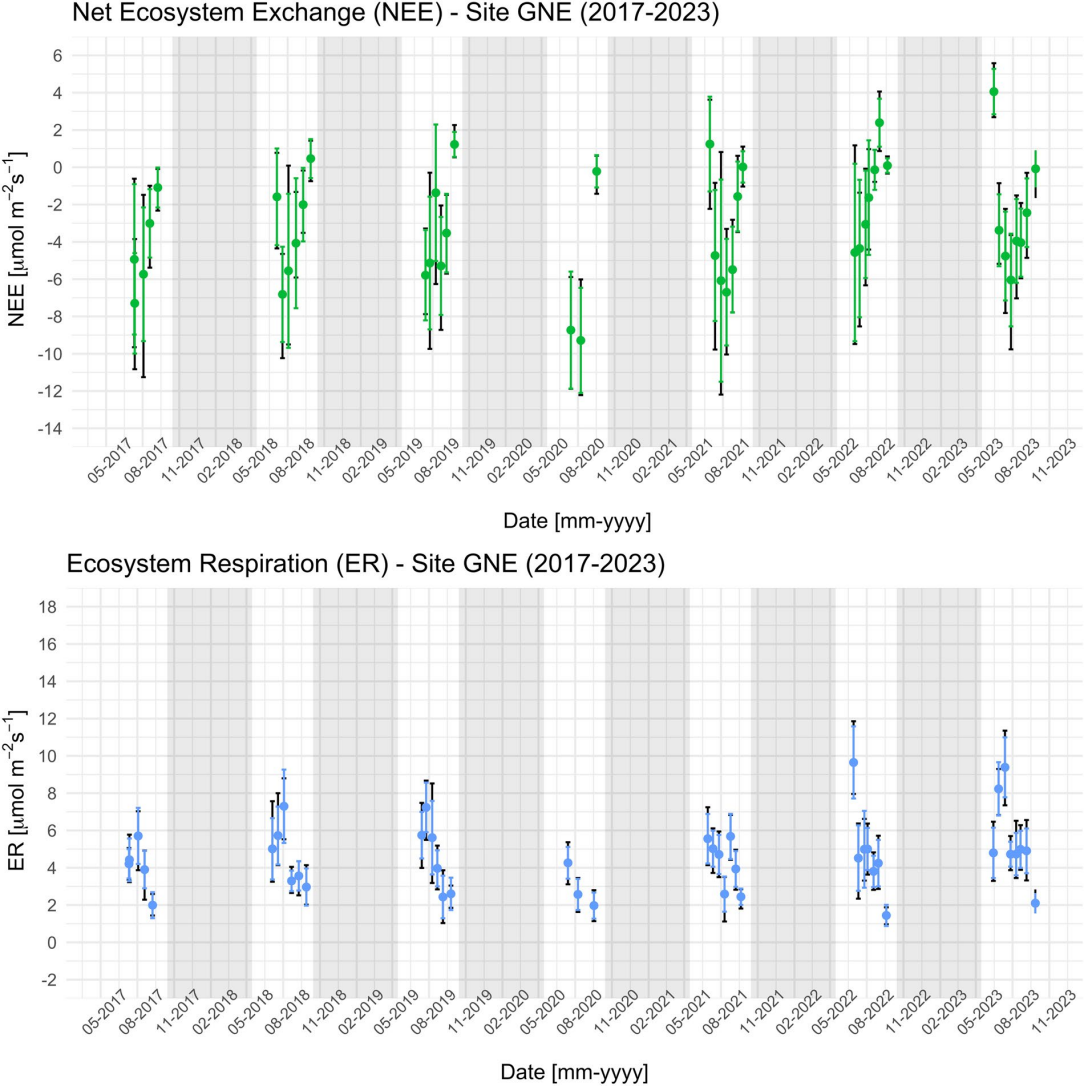
Net Ecosystem Exchange (NEE, top) and Ecosystem Respiration (ER, bottom) measured at site GNE (2017–2023). The coloured dots represent the mean values, while the dark arrows indicate the 10th and 90th quantiles. Coloured bars depict the intervals of 1 standard deviation (σ).

Mean values and variability of Net Ecosystem Exchange (NEE) and Ecosystem Respiration (ER) measured at site GNE (2017–2023) are illustrated in Fig. 5, as an example of the data from one of the five sites. Part of the NEE data discussed here have been compared with the flux estimates provided by an eddy covariance tower located at the EC site, belonging to the FLUXNET network as ICOS-Associated ecosystemic station since 2022 (IT-Niv, ref to: https://meta.icos-cp.eu/resources/stations/ES_IT-Niv). The results of the comparison indicated that the site-average of the individual NEE point measurements at the EC site were consistent with the NEE estimates provided by the eddy covariance method for the same time and date^15^.

### Meteo-climatic variables

The optimised version of the portable weather station, shown in Fig. 3, was employed starting from 2018.

During the CO_2_ flux measurements, FluxManager2 simultaneously recorded air temperature, atmospheric pressure, air relative humidity, solar irradiance, soil temperature, and soil volumetric water content (1 Hz acquisition).

Air relative humidity, air temperature, and solar irradiance were measured using LSI LASTEM thermohygrometers model DMA672.1 sheltered from direct solar radiation and LSI LASTEM pyranometers model DPA053A mounted on a portable tripod at a height of 1.5 metres above the ground^28^. The atmospheric pressure was recorded using digital barometers placed inside the flux chambers.

Soil temperature and soil volumetric water content were measured using Pt100 thermometers and Delta-T SM150T soil moisture sensors at depths of approximately 10 cm for the soil temperature and in the range of 0–5 cm for the soil moisture. The measurements were taken at about 20 cm from the collar on undisturbed soil, specifically without removing the organic layer (layer O). To account for small scale variability of soil moisture, soil volumetric water content values were also taken inside the collar area before the measurements of CO_2_ concentration to assess the moisture range (at least 3 measurements), then the probe was placed outside the collar, at a point where the soil water content was in the range of values measured inside the collar.

To ensure accuracy, these sensors were tested at CNR laboratories before and after each measurement season and calibrated in accredited laboratories every two years. The specifications for the sensors and probes are provided in Table 3.

**Table 3:** Characteristics of the sensors and probes used to measure meteo-climatic variables.

Sensor/probe & variable measured	Model & Manufacturer	Range	Accuracy	Expanded measurement uncertainty
Thermometer (AirT)	DMA672.1 LSI LASTEM Srl	[−50°:100°]	0.1 °C (@0 °C)	0.2 °C
Hygrometer (Air_RH)	DMA672.1 LSI LASTEM Srl	[0:100%]	±1% (@5:95%)	2.0%
Pyranometer (SolarRad)	DPA053A LSI LASTEM Srl	[0:2000 W m^−2^]	±1% (100:1000 W m^−2^)	2.40% [W m^−2^]
Platinum Resistance Thermometer Pt100 RTD (SoilT)	Pt100 Industrial Sensor Probe, Class B RS PRO	[−50°:100°]	0.05 °C	0.055 °C
Time-domain reflectometry (TDR) soil probe (SoilVWC)	SM150T sensor Delta-T Devices Ltd.	[0:100%]	±3.0% vol over 0%-70% vol, and at 0–60 °C	±3.0% vol over 0%–70% vol, and at 0–60 °C
Digital barometer (Pressure)	MPL3115A2 NXP Semiconductors / Freescale	[500:1100 hPa]	±0.5 hPa	±2.5 hPa

### FluxManager2

The FluxManager2 Android application (West Systems S.r.l.) is installed on a palmtop computer provided with Bluetooth; it is used to manage the instrumentation, sensors and probes and for displaying and recording the data.

FluxManager2 Android app is freely available on Google Play Store.

### FluxRevision

The FluxRevision software (West Systems S.r.l.) allows users to interpolate the CO_2_ concentration curve and calculate their slope and R^2^, using files created with FluxManager2. The software leaves the possibility to choose the linear interpolation interval.

FluxRevision is freely available for download from the West Systems website (https://www.westsystems.com/instruments/download/).

## Data Records

The dataset provided with this manuscript is organised as a comma-separated text file (.csv) and is available at the IGG-CNR-CZO Community page in the Zenodo repository^29^.

Fields are separated by semicolons and NA indicates values that are Not Available or were discarded after data quality control (ref. to the following section “Technical Validation”). Each record includes all the values of the variables recorded at each single measurement point.

Sign convention is the following: the flux from the atmosphere *to* the soil/ecosystem (e.g., photosynthetic CO_2_ uptake, GPP) is *negative*, whereas the flux *from* the soil/ecosystem (ER) to the atmosphere is *positive*. Thus, NEE = GPP + ER can be either positive or negative. NEE and ER fluxes are reported in μmolCO_2_ m^−2^ s^−1^.

Names/acronyms used in the dataset and their description are listed in Table 4. Meteo-climatic variables recorded during the measurement of NEE or during the measurement of ER bring the suffix NEE or ER respectively (i.e., Pressure_NEE = atmospheric pressure recorded during the measurement of Net Ecosystem Exchange).

**Table 4:** Description of the dataset variables.

Variable name	Unit	Description
DATE	dd/mm/yy	Campaign date in dd/mm/yy format
YEAR	yyyy	Campaign year in yyyy format
TIME_UTC + 2_NEE	H:M:S	Time of NEE measurement in UTC + 02
TIME_UTC + 2_ER	H:M:S	Time of ER measurement in UTC + 02
Site		Measurement site (AL, CARB, GLAC, GNE, EC)
LONG_(E)		Longitude East, as recorded by the integrated palmtop computer GPS (WGS84 spatial reference system)
LAT_(N)		Latitude North, as recorded by the integrated palmtop computer GPS (WGS84 spatial reference system)
ELEVATION	m	Altitude above sea level (m)
SolarRad_NEE	W m^−2^	Solar irradiance in [W m^−2^] during NEE measurement
AirT_NEE	°C	Air temperature in [°C] during NEE measurement
Air_RH_NEE	%	Air relative humidity in [%] during NEE measurement
Pressure_NEE	hPa	Atmospheric pressure in [hPa] during NEE measurement
SoilT_NEE	°C	Soil temperature in [°C] at 10 cm depth during NEE measurement
SoilVWC_NEE	%	Soil volumetric water content in the range of 0–5 cm depth in [%] during NEE measurement
FLUX_NEE	μmolCO_2_ m^−2^ s^−1^	NEE CO_2_ flux obtained through a laboratory calibration curve that allowed to convert the temporal variation of the CO_2_ concentration inside the chamber [ppm s^−1^] into the CO_2_ flux [μmolCO_2_ m^−2^ s^−1^].
SLOPE_NEE	ppm s^−1^	Slope of the linear regression of the CO_2_ concentration vs time curve [ppm s^−1^]
R2_SLOPE_NEE		R-squared of the linear regression of the CO_2_ concentration vs time curve [ppm s^−1^]
SolarRad_ER	W m^−2^	Solar irradiance in [W m^−2^] during ER measurement
AirT_ER	°C	Air temperature in [°C] during ER measurement
Air_RH_ER	%	Air relative humidity in [%] during ER measurement
Pressure_ER	hPa	Atmospheric pressure in [hPa] during ER measurement
SoilT_ER	°C	Soil temperature in [°C] at 10 cm depth during ER measurement
SoilVWC_ER	%	Soil volumetric water content in the range of 0–5 cm depth in [%] during ER measurement
FLUX_ER	μmolCO_2_ m^−2^ s^−1^	ER CO_2_ flux obtained through a laboratory calibration curve that allowed to convert the temporal variation of the CO_2_ concentration inside the chamber [ppm s^−1^] into the CO_2_ flux [μmolCO_2_ m^−2^ s^−1^].
SLOPE_ER	ppm s^−1^	Slope of the linear regression of the CO_2_ concentration vs time curve [ppm s^−1^]
R2_SLOPE_ER		R-squared of the linear regression of the CO_2_ concentration vs time curve [ppm s^−1^]

**Fig. 6:**
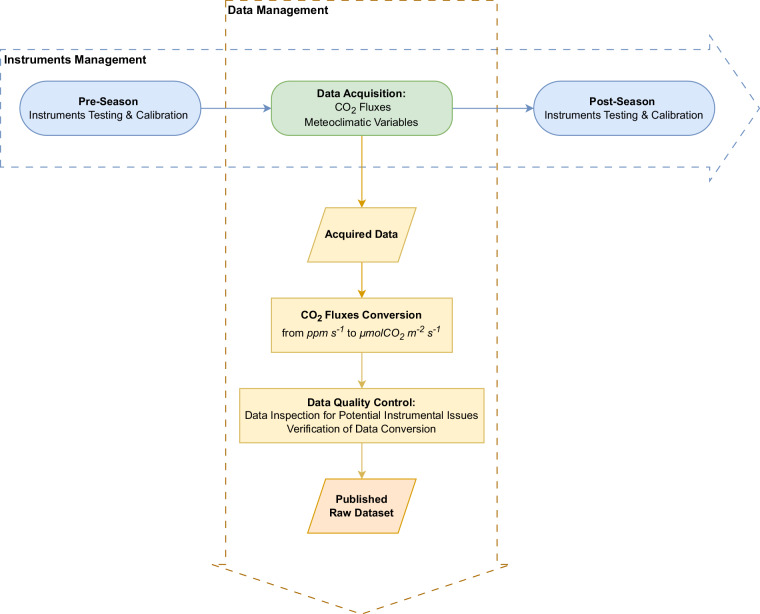
Data Management (DM) and Instruments Management (IM) workflow. The DM workflow illustrates each step from data acquisition to the final product.

A comprehensive workflow showing all the steps performed from data acquisition to the final dataset is reported in Fig. 6.

## Technical Validation

Before and after each measurement season, we tested and calibrated the instrumental equipment (including flux chamber, pump, IRGA, connecting tubes, and portable weather stations) to ensure proper functioning and performance. We use a calibration curve that is specific to the instrumental setup and is determined on a case-by-case basis to account for any variations or changes in the equipment over time.

**Fig. 7:**
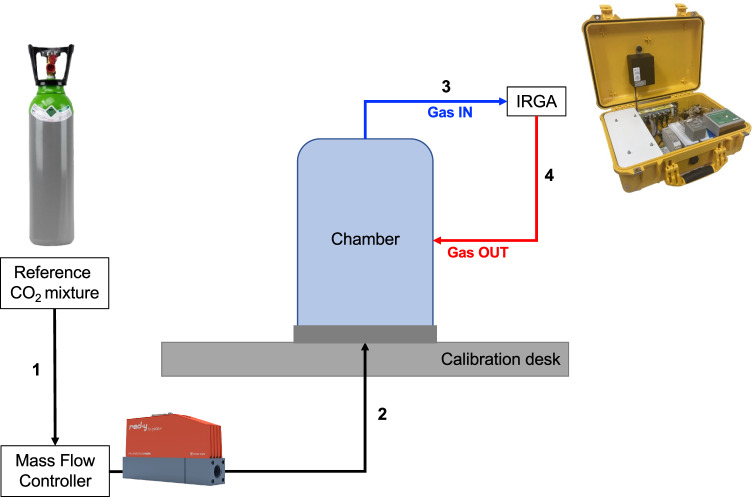
Scheme of the calibration setup. A high-precision thermal mass flow controller is used to set a constant CO_2_ mass flow (1), which is then routed inside the flux chamber through a hole in a rubber-covered desk (calibration desk) that simulates the soil surface (2). The CO_2_ mass flow then enters the flux chamber, and the air from the headspace of the chamber is pumped at a constant flow rate of 3 l/min into the IRGA (3). The IRGA is used to measure the concentration of CO_2_ in the air sample. Finally, the air sample is re-injected into the flux chamber (4).

Flux chamber calibration (Fig. 7) is conducted under controlled environmental conditions in the laboratory using reference CO_2_ mass flow rates obtained from a high-precision thermal Mass Flow Controller (MFC) specifically designed for gases (red-y smart controller GSC, Vögtlin Instruments GmbH), and high-precision CO_2_ mixtures with certified concentrations. Two different high-precision CO_2_ mixtures were used: 1) CO_2_ 2.00%mol, CH_4_ 1.00%mol, and N_2_; 2) CO_2_ 1.00%mol, CH_4_ 500 ppm mol, and N_2_. The calibration of the measurement apparatus is essential for reducing the uncertainty of CO_2_ flux estimates. Our research group has been involved in investigating the uncertainty associated with CO_2_ flux measurements, with a focus on very low fluxes, resulting in the publication of the article titled “*Non-steady-state closed dynamic chamber to measure soil CO*_*2*_ *respiration: A protocol to reduce uncertainty*”^22^. The calibration process follows the same measurement procedures used in the field and the reference CO_2_ mass flow rates were chosen to cover the range of fluxes expected in the field^30,31^ (but not exceeding two orders of magnitude^22^). To test the reproducibility of the measurements, we perform 5 to 8 replicates at each predetermined CO_2_ flux.

The laboratory tests indicated that the devices achieved good reproducibility for data acquisition times of 90 seconds. However, for very low fluxes (close to detection limit), it was necessary to increase the acquisition time up to 120–150 seconds to obtain reliable results.

**Fig. 8:**
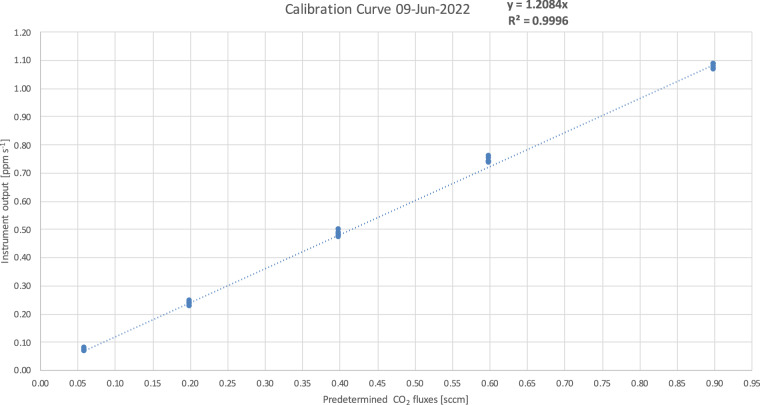
Example of a calibration curve. Predetermined CO_2_ flux values in [standard cc min^−1^] are compared with the instrument outputs in [ppm s^−1^].

Figure 8 is an example of a calibration curve. It shows the intercept of the linear interpolation of CO_2_ concentration vs time obtained with the flux chamber (in ppm s^−1^) versus the predetermined CO_2_ fluxes (in cc min^−1^).

The calibration curve is used in the conversion of the CO_2_ fluxes measured in the field. Initially, CO_2_ fluxes are corrected for the ratio between atmospheric pressure and air temperature recorded during the measurement, and those recorded in the laboratory when the calibration curve was obtained. Then, from the equation of the calibration curve, the CO_2_ fluxes are initially converted in cc min^−1^ - which is the measurement unit of the predetermined CO_2_ used in the calibration curve - and then in μmolCO_2_ s^−1^. Finally, the obtained values are divided by the collar area (0.036 m^2^) to obtain the CO_2_ fluxes in μmolCO_2_ m^−2^ s^−1^.

In addition to calibration, the IRGA were checked periodically to ensure proper operation by performing the following tests:

1. Verifying the zero CO_2_. It is verified by adding a CO_2_ scrubber to the air inlet of the IRGA and by using a zero CO_2_ cylinder in the laboratory (i.e., pure N_2_). The CO_2_ scrubber is used to reduce any atmospheric CO_2_ contamination to zero and ensure accurate readings.
2. Verifying the primary CO_2_ span by measuring concentrations of 1.000 or 10.000 ppm CO_2_.
3. Verifying the secondary CO_2_ span by measuring near-ambient levels of CO_2_.

To ensure high data quality, a data control process was conducted according to the outlined procedures. For each measurement campaign, the data were examined for anomalies or irregularities that could indicate potential instrumental malfunctions, such as battery failure. The records corresponding to the identified critical issues were not removed from the dataset, rather the corresponding fields were reported as NA (Not Available). Furthermore, the correct application of formulas and calibration curves for each campaign to convert raw data from ppm s^−1^ to μmolCO_2_ m^−2^s^−1^ was verified. This targeted approach to quality control aimed to preserve the rawness of the dataset, while addressing potential instrumental issues and thus ensuring correct data conversion.

## Data Availability

No custom code was generated for this work.
